# New techniques, new challenges—The dilemma of pain management for less invasive surfactant administration?

**DOI:** 10.1002/pne2.12033

**Published:** 2020-07-09

**Authors:** Ashanti Balakrishnan, Ranveer S. Sanghera, Elaine M. Boyle

**Affiliations:** ^1^ University Hospitals of Leicester NHS Trust Leicester UK; ^2^ Department of Health Sciences University of Leicester Leicester UK

**Keywords:** analgesia, less invasive surfactant administration, pain, premedication, preterm, sedation

## Abstract

Recent years have seen the increasing use of noninvasive respiratory support in preterm infants with the aim of minimizing the risk of mechanical ventilation and subsequent bronchopulmonary dysplasia. Respiratory distress syndrome is the most common respiratory diagnosis in preterm infants, and is best treated by administration of surfactant. Until recently, this has been performed via an endotracheal tube using premedication, which has often included opiate analgesia; subsequently, the infant has been ventilated. Avoidance of mechanical ventilation, however, does not negate the need for surfactant therapy. Less invasive surfactant administration (LISA) in spontaneously breathing infants is increasing in popularity, and appears to have beneficial effects. However, laryngoscopy is necessary, which carries adverse effects and is painful for the infant. Conventional methods of premedication for intubation tend to reduce respiratory drive, which increases the likelihood of ventilation being required. This has led to intense debate about the best strategy for providing appropriate treatment, taking into account both the respiratory needs of the infant and the need to alleviate procedural pain. Currently, clinical practice varies considerably and there is no consensus with respect to optimal management. This review seeks to summarize the benefits, risks, and challenges associated with this new approach.

## 1 INTRODUCTION

Historically, pain in newborn babies has been under‐recognized and, consequently undertreated.^1^ It was not until the late 1980s that seminal studies of babies undergoing surgery convincingly demonstrated that even preterm neonates are sufficiently developed, both anatomically and physiologically, to experience and respond to noxious stimuli.^2^, ^3^ Moreover, these studies showed that the responses to noxious stimuli could be attenuated by the use of anesthesia and analgesia.^4^ Prior to this, infants had been, for many years, subjected to major surgery with no, or minimal anesthesia and without postoperative analgesia. Still, it is probably fair to say that clinicians were slow to take this message on board.

There has been ongoing debate about whether receiving mechanical ventilation is a painful experience, but what is not any longer questioned is that the process of endotracheal intubation is painful and stressful.^5^ We cannot know exactly how infants subjectively perceive pain; the lack of self‐report in preverbal infants means that we can only extrapolate from information available to us about how older individuals experience different procedures. Endotracheal intubation is rarely performed in adults without the aid of analgesia and/or sedative medication. However, there are reports of necessary “awake” intubations in adults. Such a report describes the patient's experience of the procedure: “It felt like I could not swallow, like I would suffocate on the fluid, like as I cough and throw up at the same time and then it burns”.^6^ This graphic description leaves the reader in no doubt that the procedure can be unpleasant. Neonates were routinely subjected to airway management and intubation without sedative drugs until relatively recently. A number of research studies have considered the use of premedication for intubation in the newborn population. A retrospective review found that the use of premedication facilitated intubation for operators of all levels of experience.^7^ Randomized placebo‐controlled trials are few and have enrolled only small numbers of babies. Results are conflicting, with some demonstrating fewer attempts^8^, ^9^ and reduced time to intubate with premedication, and another unable to show no benefit with the use of morphine versus placebo. There have also been multiple studies comparing different drugs and combinations of drugs that have not included a placebo group.^10^, ^11^, ^12^, ^13^, ^14^, ^15^ While acknowledging the limitations in evidence and gaps in knowledge in this area, premedication has long been recommended for neonates undergoing intubation in all but emergency situations.^16^

It had taken many years of research and education to reach a stage where the majority of neonatal units in developed countries had adopted the routine use of premedication for intubation, although practice has been varied with respect to the medication used.^17^ However, in recent years, manipulation of the airway in “awake” preterm neonates is again becoming accepted practice. How can this be, when evidence has pointed to better outcomes with the use of premedication? Less invasive surfactant administration (LISA) (also sometimes referred to as minimally invasive surfactant therapy (MIST)) has started to be used more readily, with more and more units adopting this approach.^18^ This involves the insertion of a fine catheter through the vocal cords of a spontaneously breathing infant, usually while maintaining noninvasive respiratory support during the procedure. Surfactant is administered through this catheter, which is removed immediately after to allow the baby to continue non‐invasive respiratory support. With the advent of this type of procedure, controversy has once again arisen around the concept of giving sedation and analgesia. We aim to disentangle the reasons behind what, to some, may appear to be a retrograde step in the management of pain and discomfort in this population, yet to others, may represent a very significant advance in respiratory management.

## 2 PREMEDICATION FOR LISA/MIST: THE CASE FOR

The goal of LISA/MIST procedures is to reduce the length of time spent on mechanical ventilation, the adverse effects of positive pressure ventilation, and bronchopulmonary dysplasia (BPD). A systematic review and meta‐analysis, of minimally invasive surfactant administration versus intubation and mechanical ventilation, was performed by Aldana‐Aguirre et al^19^ This showed that spontaneously breathing infants, when treated for respiratory distress using LISA/MIST, had a reduced need for mechanical ventilation and a reduction in the composite outcome of BPD or death at 36 weeks of gestation. As studies have suggested benefit in this way,^20^ LISA/MIST has become the treatment of choice in infants with respiratory distress in whom surfactant therapy is deemed to be indicated and where it is thought that mechanical ventilation might safely be avoided.^21^

Yet, advances in respiratory management and avoidance of intubation bring with them new challenges, including the dilemma of how to manage pain associated with the unavoidable procedure of laryngoscopy.^22^ Laryngoscopy is necessary for LISA/MIST, in order for the surfactant to be successfully delivered through the vocal cords. It is accepted that laryngoscopy is a painful procedure for neonates and is associated with adverse effects. Stretching of the pharynx can cause parasympathetic and sympathetic responses of bradycardia and pulmonary hypertension as a protective airway reflex.^5^ Infants appear disturbed by, and commonly resist the insertion of the laryngoscope, which can lead to an increase in intracranial pressure; impairment in the venous return to the brain can cause intracranial hypertension, which, in turn, could lead to the possibility of intraventricular hemorrhage (IVH).^16^, ^23^, ^24^, ^25^, ^26^, ^27^

In order to counteract the noxious stimulus of laryngeal stimulation for intubation in neonates, it has become common practice to use premedication for analgesia and sedation in elective and non‐emergency intubations. Does the administration of surfactant using a small catheter constitute “intubation,” or can it be managed differently? As LISA/MIST involves the same unpleasant direct laryngoscopy or video‐laryngoscopy for visualizing the vocal cords in order to correctly site the small catheter, it has been argued that similar premedication should be used. This is because laryngoscopy has long been regarded as a painful part of the intubation procedure.^28^ Premedication for sedation and analgesia before neonatal intubation has been addressed in a number of studies and publications in an attempt to determine the safest and most effective strategies and the most appropriate drugs.^29^, ^30^, ^31^, ^32^ Trials of premedication for LISA/MIST have been fewer. Dekker et al measured ComfortNeo scores when using propofol sedation for MIST, both in an observational study and in a randomized trial, and showed that it reduced scores.^23^, ^33^ Descamps et al also studied propofol in their retrospective review and showed only mild and transient adverse effects.^32^ Bourgoin et al showed reduced pain responses with a combination of ketamine and atropine.^34^ However, while studies have shown that premedication is helpful for reducing pain and discomfort, no one drug or combination of drugs has been shown to be superior, guidance varies, and none is universally used.

Neonatal exposure to painful stimuli at a critical time of brain development in preterm infants is known to be associated with long‐term problems for graduates of neonatal intensive care.^35^, ^36^, ^37^, ^38^ Schwaller et al discussed evidence from animal models about mechanisms by which pain and stress may result in changes in nociceptive pathways and that painful procedures may cause a decrease in the development of white matter and subcortical gray matter.^39^ This may lead to hyperalgesia, and there is evidence that it can contribute to chronic pain states in the future.^39^ Perlman and Volpe showed that routine endotracheal suctioning changes cerebral blood flow, suggesting that this is a stressful procedure for the infant.^40^ It can be assumed that laryngoscopy would also be likely to cause similar effects. She proposes that a preterm infant's response to pain can lead to changes in the hypothalamic‐pituitary‐adrenal (HPA) axis, leading to the production of abnormal amounts of cortisol. It has also been suggested that this can cause an atypical pro‐inflammatory response and a long‐term immune cell activation.^41^ Grunau suggests that pain and stress may not only hinder body and head growth but may also cause poor brain development, reduced pain thresholds and disruption of the set points of biological circuits.^41^ It is plausible therefore that the procedure of laryngoscopy, which causes immediate pain and stress for the neonate, may also contribute to the adverse effects of recurrent pain in later life.

In addition to the physiological evidence, it may seem unreasonable, from the humane and ethical standpoint to ignore the potential for pain and withhold premedication. Should small, fragile infants who are physically unable to resist this “assault” be subjected to laryngoscopy without the kind of analgesia and sedation that would be given as part of usual management in children or adults? This is even more pertinent when considering levels of experience of the clinicians performing the procedure. In many neonatal units, the “front‐line” practitioners who perform common procedures are generally the more junior clinicians, often with limited experience of even conventional intubation methods. Routine use of premedication for intubation has become commonplace over a number of years, and many of these clinicians will not have developed the skills needed to swiftly perform the procedure without first giving sedation, analgesia, and muscle relaxation. It is recognized that failed attempts at intubation are regular occurrences.^42^ Premedication has been shown to increase the success rate of intubation.^8^ De Kort et al investigated the success rate of LISA without sedation and found that there was a low success rate on the first attempt of LISA without sedation.^43^ De Kort and others have suggested that, with premedication, success rates could be improved.^31^, ^43^, ^44^ If LISA/MIST procedures, like intubation for ventilation, are often to be performed by the least experienced personnel, then premedication could be seen as vital to avoid multiple attempts, as well as associated oral and airway trauma and long‐term adverse effects. Videolaryngoscopy is increasingly used as a teaching aid and has shown promise on the neonatal unit as a means of facilitating rapid and safe intubation and delivery of surfactant by LISA.^45^ Future studies might usefully explore the combined use of videolaryngoscopy and premedication for LISA/MIST.

## 3 PREMEDICATION FOR LISA/MIST: THE CASE AGAINST

Preterm neonates are known to be at risk of developing BPD and long‐term respiratory disease.^46^ Although the development of chronic lung disease in preterm infants is multifactorial, a predisposing factor is prolonged mechanical ventilation. Therefore, in preterm newborn infants with respiratory problems, non‐invasive ventilation strategies, such as continuous positive airway pressure (CPAP), have now become the preferred method of early respiratory support with the aim of reducing the incidence and severity of BPD. However, respiratory distress syndrome (RDS) caused by surfactant deficiency contributes significantly to CPAP failure.^24^ In spontaneously breathing preterm infants with RDS receiving nasal CPAP, LISA/MIST has been described as an alternative to endotracheal intubation for surfactant administration.^19^, ^32^ LISA aims to provide the appropriate dose of surfactant while the infant is breathing spontaneously.^19^ LISA is different from other modes of surfactant delivery as it allows the infant to maintain the function of the glottis to continue breathing, and use the physiological function of the larynx by using a very thin catheter for surfactant application without the almost complete obstruction by a larger endotracheal tube. Such small tubes allow adduction of the vocal cords. Following LISA, because the infant is breathing spontaneously, surfactant spreads quickly, making use of its unique biophysical properties without the need for positive pressure ventilation_._ ^24^, ^47^, ^48^

LISA is reported to reduce the risk of mechanical ventilation in randomized controlled trials.^19^ A systematic review showed that avoiding mechanical ventilation also has the potential to decrease the combined risk for BPD and death.^23^, ^49^ However, the current technology for LISA still demands laryngoscopy with all its unpleasant effects for infants.^19^ Although sedation for endotracheal intubation of infants is widely adopted, there is no consensus about whether sedation should be used for LISA.^19^

Klotz et al's European survey, sent to 324 neonatologists from different centers within 37 European countries between December 2015 and March 2016, indicated that the percentage of centers using LISA increased, but with wide variation in procedure. In particular, there was no consensus about whether sedation should be used.^50^ Jeffreys et al's recent UK Survey sent to 196 neonatal units between May and July in 2018 on LISA practices across all neonatal units showed that 49% of units that used LISA did not use sedation/premedication.^18^ The most recent UK survey of practice reported that LISA has been slow to have been adopted in neonatal units.^51^ In those that use LISA, fentanyl is most often the premedication of choice, but 14.5% reported not using any form of sedation. Opioids, along with atropine, are among the most commonly used drugs for LISA.^18^, ^50^, ^51^ International guidelines recommend sedation for intubation but it can hamper spontaneous breathing, which is necessary for LISA.^32^ This variation in practice begs the question of whether we should be routinely giving sedation?

Germany was one of the first few centers to adopt LISA into their clinical practice, and so the German Neonatal Network (GNN) has participated in several pivotal trials of LISA.^47^, ^48^ This has resulted in a cohort of more than 5000 infants treated with LISA. In Germany, the majority of surfactant treatments are now performed by LISA.^24^, ^47^, ^48^ Of note is the fact that most centers in Germany attempted LISA in infants born at <26 weeks of gestation without using analgesic medication, at least for the first attempt. Instead, nonpharmacological methods of analgesia such as positioning, holding, “facilitated tucking,” and/or sucrose solutions were used.^24^, ^47^ In one study in which surfactant was given to spontaneously breathing infants, analgesic or sedative drugs were used in 26% of infants at the discretion of the attending physician.^47^ Administration of surfactant using LISA has become more widely practised in neonatal intensive care units around the world and has become an acknowledged alternative to the standard way of delivering surfactant, but the need for sedation/analgesia for LISA is still a subject for debate.^23^

This great variation in practice stems from the fact that, while sedation might increase the chance for an uneventful, smooth, and successful procedure, it also has the propensity to compromise the infant's respiratory drive, which is a prerequisite for successful LISA/MIST. Since respiratory depression is an indication for mechanical ventilation, this would seem to be counterproductive. This is seen in Dekker et al's randomized controlled trial of sedation during MIST, which showed that low‐dose sedation increased comfort during the MIST procedure in preterm infants.^33^ However, this was accompanied by a greater risk of desaturation (SpO_2_ < 85%) events during the procedure in the group receiving even a low dose of propofol (91% vs 69%, *P* = .023). Not surprisingly, with a greater risk of oxygen desaturation, there was an increased need for nasal intermittent positive pressure ventilation (93% vs 47%, *P* < .001).^23^

The salient question then becomes, if LISA is to be performed without premedication, are we still, however, inflicting pain? Recent studies have shown that procedural pain can affect neurodevelopment as the exposure to multiple painful procedures can lead to ongoing stress.^41^, ^52^, ^53^, ^54^ There is also evidence that these adverse events can be prevented or minimized by using both pharmacological and nonpharmacological interventions during painful procedures.^55^, ^56^ This then implies that these long‐term negative effects can be negated with the avoidance of stress and pain whenever possible. However, drugs used for stress/pain relief also have both acute and long‐term side effects.^57^, ^58^, ^59^ A high level of neonatal analgesia correlates strongly with reduced cerebellar volume and poorer cognitive and motor outcomes in infancy.^60^

A variety of drugs have been studied for the purpose of analgesia/sedation during the procedures of INSURE (INtubation, SURfactant, Extubation) or LISA, with fentanyl, ketamine, and propofol being the most frequently used medications.^24^, ^47^ However, no drug is without unwanted adverse effects, and studies indicate that premedication may help to reduce pain scores, but may interfere with spontaneous breathing especially in preterm infants, who are even more sensitive to these effects.^23^, ^24^ Reported experience from a retrospective review comparing MIST with and without propofol sedation showed a significant difference in ComfortNeo scores between the groups.^23^ Others have shown no difference or a reduction in complications or long‐term effects.^23^, ^48^, ^61^, ^62^

LISA requires specific skills and should therefore only be performed by experienced neonatal clinicians. It has been reported that very few infants demonstrate discomfort while surfactant is being instilled via LISA and, if symptoms occur, the administration (usually over <2 minutes) can be slowed down.^24^ If the reason for sedation is to improve success rates of LISA, then studies have shown that failure to insert the catheter through the vocal cords at first attempt, significant surfactant reflux, acute desaturations, bradycardia, and/or need for manual ventilation have been observed during LISA/MIST manipulations.^23^, ^24^, ^63^ Such complications were reported to have the highest incidence in studies that treated more mature premature infants several hours after birth, and where CPAP was interrupted during LISA.^24^, ^61^, ^62^, ^63^ To tackle this challenge, it is suggested that the key is to treat early with continued CPAP and use a gentle approach with laryngoscopy to avoid discomfort as much as possible. There are some studies underway looking at continuous monitoring of saturation and cerebral saturation by near‐infrared spectroscopy, which indicate that a careful direct laryngoscopy technique is important to avoid the complications described.^64^

Opioid medications are often employed for premedication and analgesia on the neonatal unit.^65^ Long‐term adverse neurodevelopmental effects following the neonatal use of opioids have been reported, mainly with continuous or repeated use in preterm populations rather than single doses, but results between studies have been conflicting.^60^, ^66^, ^67^, ^68^, ^69^, ^70^, ^71^ Potential early adverse effects on breathing and blood pressure are well described.^58^, ^72^ Squillaro et. al's recent publication asks the question of whether we should be managing neonatal procedural pain using an opioid‐sparing approach, thereby eliminating the risk of affecting the infant's respiratory drive.^73^ They reviewed 3 different options for pain control: (a) nonopioid pharmacological agents (acetaminophen, NSAIDs, dexmedetomidine, and gabapentin); (b) local and regional anesthesia (spinals, epidurals, subcutaneous injections, and topical anesthesia); and (c) nonpharmacological alternatives (skin‐to‐skin care, facilitated tucking, sucrose, breastfeeding, and non‐nutritive sucking). The conclusions were that opioid‐sparing agents can provide pain control, and may usefully replace opioid analgesia, or be used as adjunctive therapies to reduce opioid exposure. Nonpharmacological alternatives used alone or in combination with other interventions may help to alleviate mild‐to‐moderate pain and decrease neonatal distress during painful procedures.^73^ However, this approach has not yet been specifically tested for laryngoscopy.

## 4 CONCLUSIONS

It is challenging to find the delicate balance between over‐ and under‐treatment of neonatal pain, as both inadequate pain control and excess opioid use have potential adverse effects and have been associated with poor developmental outcomes. However, 2‐year outcomes reported very recently from a randomized controlled trial are reassuring about long‐term safety.^74^ The introduction of new procedures and techniques is important to advance management and prevention of common and devastating diseases related to prematurity, such as BPD. However, it is important that, in advancing one area of neonatal care, we do not inadvertently undermine another important area. Neonates are unable to articulate their discomfort and pain, and it is incumbent on us to minimize their distress in the safest and most effective way, while optimizing respiratory management. It is likely that the optimal approach to this dilemma in new respiratory support techniques such as noninvasive respiratory support and the use of LISA/MIST has not yet been determined. Few studies to date have directly assessed pain and distress in infants during LISA/MIST procedures, and this should be a priority, but will require careful consideration of the ethical issues involved.^75^ There is a need to clarify, through rigorous research, both risks and benefits associated with this procedure. These studies should include the evaluation of different approaches, whether pharmacological or nonpharmacological, to enhance comfort during the procedure in order to achieve the balance we are seeking, so that infants can benefit from the entire body of evidence in all areas of neonatal care.

## CONFLICT OF INTEREST

There are no conflicts of interest to disclose.
